# Transient Replication in Specialized Cells Favors Transfer of an Integrative and Conjugative Element

**DOI:** 10.1128/mBio.01133-19

**Published:** 2019-06-11

**Authors:** François Delavat, Roxane Moritz, Jan Roelof van der Meer

**Affiliations:** a Department of Fundamental Microbiology, University of Lausanne, Lausanne, Switzerland; CEH-Oxford

**Keywords:** chromosome replication, horizontal gene transfer, *Pseudomonas putida*, TraI relaxase, adaptation, fitness, origin of transfer, single-cell studies, time-lapse microscopy

## Abstract

Integrative and conjugative elements (ICEs) are widespread mobile DNA within bacterial genomes, whose lifestyle is relatively poorly understood. ICEs transmit vertically through donor cell chromosome replication, but in order to transfer, they have to excise from the chromosome. The excision step makes ICEs prone to loss, in case the donor cell divides and the ICE is not replicated. By adapting the system of LacI-cyan fluorescent protein (CFP) binding to *lacO* operator arrays, we analyze here the process of excision and transfer of the ICE for 3-chlorobenzoate degradation (ICE*clc*) in individual cells of the bacterium Pseudomonas putida. We provide evidence that ICE*clc* excises exclusively in a subset of specialized transfer-competent cells. ICE*clc* copy numbers in transfer-competent cells were higher than in regular nontransferring cells but were reduced in mutants lacking the ICE *oriT1* origin of transfer, the ICE DNA relaxase, or the excision recombination sites. Consistently, transfer-competent cells showed a higher proportion without any observable LacI-CFP foci, suggesting ICE*clc* loss, but this proportion was independent of the ICE relaxase or the ICE origins of transfer. Our results thus indicated that the excised ICE becomes transiently replicated in transfer-competent cells, with up to six observable copies from LacI-CFP fluorescent focus measurements. Most of the observed ICE*clc* transfer to ICE-free P. putida recipients occurred from donors displaying 3 to 4 ICE copies, which constitute a minority among all transfer-competent cells. This finding suggests, therefore, that replication of the excised ICE*clc* in donors is beneficial for transfer fitness to recipient cells.

## Abstract (precis)

Bacterial evolution is driven to a large extent by horizontal gene transfer (HGT)—the processes that distribute genetic material between species rather than by vertical descent. The different elements and processes mediating HGT have been characterized in great molecular detail. In contrast, very little is known on adaptive features selecting HGT evolvability and fitness optimization. By studying the molecular behavior of an integrated mobile DNA of the class of integrative and conjugative elements in individual Pseudomonas putida donor bacteria, we report here how transient replication of the element after its excision from the chromosome is favorable for its transfer success. Since successful transfer into a new recipient is a measure of the element’s fitness, transient replication may have been selected as an adaptive benefit for more-optimal transfer.

## INTRODUCTION

Integrative and conjugative elements (ICEs) are pervasive and permissive infestations of bacterial genomes (1–3). Not unlike prophages, but differently from plasmids, ICEs display a dual lifestyle. Most cells in a population maintain the ICE chromosomally integrated, but under specific conditions a small proportion (estimated to be between 1 in 10^2^ and 1 in 10^7^ cells depending on the ICE [3]) excises the ICE and produces an extrachromosomal ICE DNA molecule (1–3). The excised ICE molecule can transfer into a recipient cell by conjugation, where it subsequently reintegrates. ICEs have attracted considerable interest because they frequently transfer and integrate into a wide taxonomic range of hosts and carry gene functions of potential adaptive benefit to the host, such as genes coding for antibiotic or heavy metal resistance, plant symbiosis, or xenobiotic compound metabolism (4, 5).

**FIG 1:**
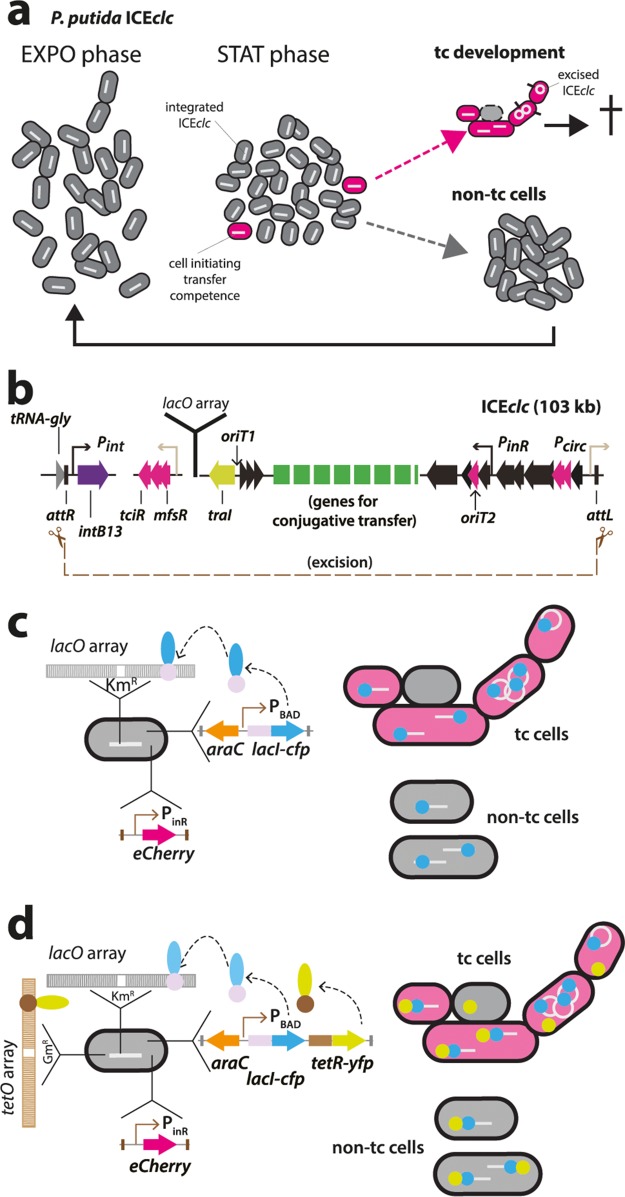
Principle of ICE*clc* detection in individual and transfer-competent Pseudomonas putida cells. (a) ICE*clc* remains integrated and silent in exponentially growing cells (EXPO, white bars in gray cells). Three to 5% of cells under nongrowing conditions (STAT) activate the core ICE promoters for its transfer competence program (magenta cells). Upon new nutrient addition, the transfer-competent cells (tc) excise (white circle) and transfer the ICE (black protrusions from cells). tc cells are impaired for cell division and frequently lyse (dashed cell outline). Non-tc cells continue to divide normally in exponential phase. (b) Schematic outline of ICE*clc*, its recombination boundaries, positions of genes mutated in this study, and insertion position of the *lacO_ARRAY_*. (c and d) LacI-CFP single or LacI-CFP/TetR-YFP double fluorescent focus formation by ectopic expression of a single-copy chromosomally inserted arabinose-inducible *lacI-cfp* or *lacI-cfp/tetR-yfp* gene construct. Fluorescent protein binding to the 240-fold-copied cognate DNA binding site results in visible foci, as illustrated schematically. Cells are colabeled with a single-copy chromosomally inserted fusion of *echerry* to the P_inR_ promoter of ICE*clc*, which is active exclusively in tc cells. tc cells losing the ICE upon excision are depicted in gray.

The model we use here is ICE*clc*, an element originally discovered in Pseudomonas knackmussii B13, which bestows on its host a xenometabolic pathway to grow on 3-chlorobenzoate (3-CBA) (6–8). Several characteristics of ICEs from the ICE*clc* family contribute to their remarkable ecological success in colonizing a large diversity of bacterial genomes (7). In its integrated form, ICE*clc* is replicated with the host genome and remains largely without fitness cost to the host (9, 10). Although silent in exponentially growing cells, the ICE*clc* genes for horizontal transfer start to be expressed when all 3-CBA substrate in culture is depleted, turning some 3 to 5% of stationary-phase cells into a subset of specialized transfer-competent (tc) cells (Fig. 1a) (11–13). The ICE does not excise or transfer at this point but does so only when tc cells are activated with fresh nutrients (Fig. 1a) (14). Active tc cell donors for the ICE further distinguish themselves from non-tc cells by their reduced capacity for cell division and frequent lysis (12). The poor reproduction from tc cells has only limited effects on population fitness because of their small subpopulation size (14).

Despite their restricted capacity to divide, tc cells are on average highly effective in transferring ICE*clc* (14). This suggested a number of specific adaptations favoring ICE transfer fitness, such as the previously demonstrated induced formation by ICE*clc* of small tc cell groups that have an increased chance to contact recipients (15). A particularly critical moment for ICE fitness, however, is when the ICE is excising from the chromosome and engages in further conjugative steps. Quantitative PCR (qPCR) data from other ICE models in Bacillus subtilis and Vibrio cholerae have suggested that ICEs transiently replicate after excision, which increases the probability for the ICE to be maintained in dividing daughter cells (16–18). These studies, however, were population based and did not take individual cell fates into account. Extrachromosomal replication of ICE*clc* has not been studied so far, but in contrast to what is known from B. subtilis and V. cholerae, ICE*clc*-induced tc cells of *P. knackmussii* (12) or Pseudomonas putida do not contribute to the reproductive success of the population (14). Furthermore, previous single-cell studies on ICE*clc* in P. putida have suggested that individual tc cell donors can transfer the ICE to 2 to 3 surrounding recipient cells, the mechanism of which remains elusive (14). Our goals were therefore to study the hypothesis that ICE*clc* is also replicated upon excision but exclusively in tc cells and serves to increase ICE transfer fitness rather than or in addition to ICE maintenance in the donor cell. Instead of relying on quantitative PCR measurements of averaged copy numbers in P. putida wild-type or mutant ICE populations, we decided to adapt and deploy molecular imaging methods (19) that would permit us to quantify ICE copy numbers in individual cells, to differentiate among tc and non-tc cell groups, and to follow ICE transfer to ICE-free recipients at the single-cell level.

## RESULTS

### Visualizing ICE*clc* DNA in individual cells.

In order to distinguish and quantify single copies integrated from excised ICE*clc* DNA molecules in individual cells, we deployed the principle of fluorescent LacI-cyan fluorescent protein (CFP) fusion protein binding to a multicopy integrated *lacO* array (19). The *lacO_ARRAY_* was inserted in a single integrated copy of the ICE*clc* in the genome of P. putida (Fig. 1b and c). This strain was further tagged by an eCherry fluorescent reporter expressed uniquely in tc cells (20). In addition, we constructed strains carrying an additional *tetO_ARRAY_* near ICE*clc* on the chromosome that can be bound by ectopically expressed fluorescent TetR-yellow fluorescent protein (YFP) (Fig. 1d). In normally replicating (non-tc) cells with integrated ICE*clc*, we expected to observe 1 to 2 foci of LacI-CFP alone (when using the *lacO_ARRAY_* alone) and overlapping TetR-YFP foci (when using cells with both integrated arrays). Upon ICE*clc* excision and consequent independent replication, we expected to see 3 or more fluorescent foci and potentially larger distances between TetR-YFP and LacI-CFP foci, exclusively in tc cells (Fig. 1c and d).

**FIG 2:**
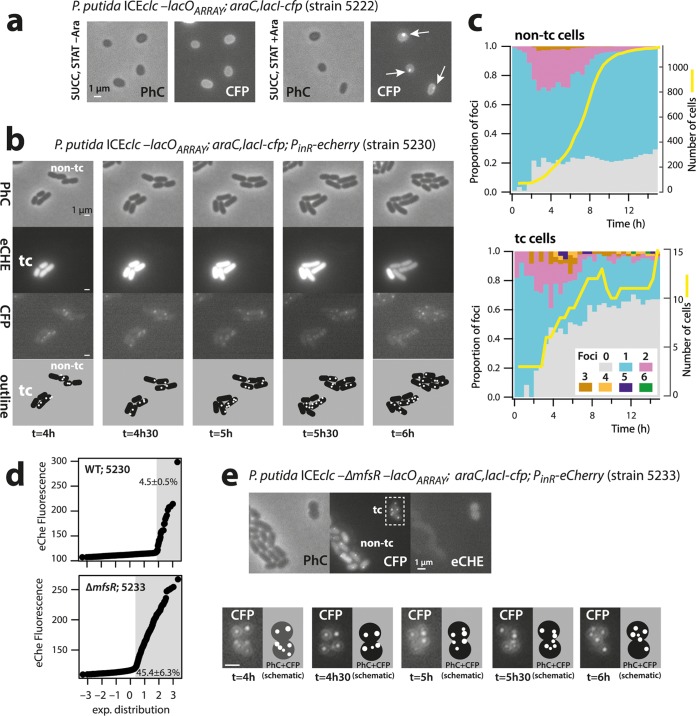
Increased ICE*clc* LacI-CFP focus numbers in transfer-competent cells. (a to e) CFP foci visible in P. putida stationary-phase cells upon arabinose induction (a) and in tc and non-tc microcolonies growing on agarose minidisks (note the stage of 6 foci in tc cell at *t* = 5 h) (b). Proportional focus distributions among growing non-tc and tc cell populations (c), with the initial discrimination of the tc cell subpopulation based on eCherry fluorescence (d). Note how the tc subpopulation increases from 4.5% in wild-type ICE*clc* to 45.4% in cells carrying ICE*clc* with a deletion of Δ*mfsR*. Dynamic focus positioning in tc cells of P. putida ICE*clc*-Δ*mfsR* (e). Bars, 1 μm.

P. putida containing wild-type ICE*clc* tagged with the *lacO_ARRAY_* ectopically expressing LacI-CFP visibly showed a clear CFP focus in individual nongrowing cells but not when *lacI-cfp* was not induced (strain 5222, Fig. 2a). These cells are in stationary phase, and the single observed fluorescent focus is thus in agreement with a single chromosomal integrated ICE*clc* copy, formed by the LacI-CFP proteins attached to the *lacO_ARRAY_*. Foci were not visible in control P. putida strains with ICE*clc* but expressing only LacI-CFP, nor in P. putida with ICE*clc* and *lacO_ARRAY_* but without LacI-CFP (see Fig. S1 in the supplemental material).

10.1128/mBio.01133-19.2 FIG S1 Specificity of LacI-CFP fusion formation in P. putida cells. P. putida with ICE*clc* and expressing *lacI-cfp* but without *lacO_ARRAY_*, or with ICE*clc* and integrated *lacO_ARRAY_* but without ectopic insertion of *lacI-cfp*, does not produce visible CFP foci in stationary phase (STAT), neither in the absence of nor in the presence of added l-arabinose (Ara). Cells imaged 24 h after inoculation on minimal medium with 10 mM succinate (SUCC) as sole carbon source in phase contrast (PhC) or in epifluorescence with CFP filter (CFP). Note that the slight fluorescence “ring” shapes even in the absence of LacI-CFP (as in strains 5214 and 5216) are due to iron siderophore production. Download FIG S1, PDF file, 1.4 MB. Copyright © 2019 Delavat et al. 2019 Delavat et al. This content is distributed under the terms of the Creative Commons Attribution 4.0 International license.

To follow the ICE*clc* in dividing tc and non-tc cells, we deposited cells of a 3-CBA-grown preculture of P. putida ICE*clc*-*lacO_ARRAY_*; *araC*, *lacI-cfp*; P_inR_-*echerry* (strain 5230, Table S1) on small agarose growth disks (14). Cells grow exponentially to microcolonies (Fig. 2b) and attain stationary phase after some 12 h (Fig. 2c, yellow lines). Importantly, because the seeding culture originates from stationary phase on 3-CBA, the population at the start of the experiment is composed of both tc and non-tc cells (Fig. 2b). These can be differentiated in the first time-lapse frame based on the eCherry fluorescence expressed from the chromosomally integrated P_inR_ reporter, which is active exclusively in tc cells (13, 20). On average, 4.5% ± 0.5% of individual cells in culture of strain 5230 were representative for tc cells (Fig. 2d). It should be noted that the criterion of higher eCherry expression (as in Fig. 2d) is sufficient to classify cells into the tc cell category (21) but not sufficiently exclusive to categorize (the other) cells as being non-tc, because some (true) tc cells may display low eCherry levels. For this reason, individual cells were further excluded from the non-tc class when their total number of offspring was less than eight. This criterion is based on the previously observed impaired cell division in tc cells (12).

10.1128/mBio.01133-19.7 TABLE S1 Strains used in this study. Download Table S1, PDF file, 0.1 MB. Copyright © 2019 Delavat et al. 2019 Delavat et al. This content is distributed under the terms of the Creative Commons Attribution 4.0 International license.

ICE*clc* copy numbers in individual cells were inferred from the number of fluorescent foci detected by using automated procedures implemented in SuperSegger (22). Foci were thresholded on the basis of score and intensity (see Materials and Methods) to discard spurious foci. The lower threshold was set such that (i) no foci were detected in P. putida with ICE*clc* and LacI-CFP but without *lacO_ARRAY_* (Fig. S1), (ii) the maximum number of foci in P. putida (*lacI-CFP*, *lacO_ARRAY_*) with ICE*clc* deleted for the *attL* recombination site was two (Fig. S2), and (iii) the maximum allowed number of foci was 8 per cell. This last criterion was based on hybridization densitometry calculations of the proportion of excised ICE*clc* in stationary-phase culture (11). As a consequence of the conservative thresholding procedure, a proportion of cells contained no detected foci (Fig. S2). Replica plating of colonies (*n* = 100) grown on plates with succinate onto agar with only 3-CBA as carbon and energy source resulted for all P. putida ICE*clc* derivative strains (Table S1) in clear growth, suggesting maintenance of the ICE at frequencies above 99.5%. Therefore, the absence of detectable foci in individual cells cannot be *a priori* interpreted as loss of ICE*clc* but is a consequence of the focus thresholding procedure.

10.1128/mBio.01133-19.3 FIG S2 Effect of score and intensity thresholding on focus distributions in P. putida UWC1*-clc5*, *ΔmfsR*, *ΔattL*, *lacO_ARRAY_*, Tn*7 araC*;*lacI-cfp*, Tn*5 P_inR_-echerry* (strain 5357). Since this strain lacks the ICE attachment sites, it cannot excise, which was verified by PCR (4). The lowest threshold setting with minimal focus appearance of >2 (score = 6 and intensity = 6) was thus used for the experiments. Download FIG S2, PDF file, 1.8 MB. Copyright © 2019 Delavat et al. 2019 Delavat et al. This content is distributed under the terms of the Creative Commons Attribution 4.0 International license.

### ICE*clc* excises and replicates in tc cells.

The majority of non-tc cells of P. putida strain 5230 with wild-type ICE*clc* at any time point during growth displayed a single LacI-CFP focus (Fig. 2c, cyan stacked bars). During exponential growth (2 to 8 h after inoculation, yellow line in Fig. 2c, non-tc cells), some 20 to 30% of cells showed two foci (Fig. 2c, magenta stacked bars). Two foci are in agreement with dividing cells replicating their chromosomal DNA, which, at some point, will thus have two ICE*clc* copies (producing two foci) before the chromosomes segregate among the daughter cells (non-tc cell micrographs in Fig. 2b). A small proportion of cells (1 to 3%, Fig. 2c) in the non-tc population displayed three CFP foci during exponential phase, which may be due to insufficient conservative thresholding (Fig. S2).

**FIG 3:**
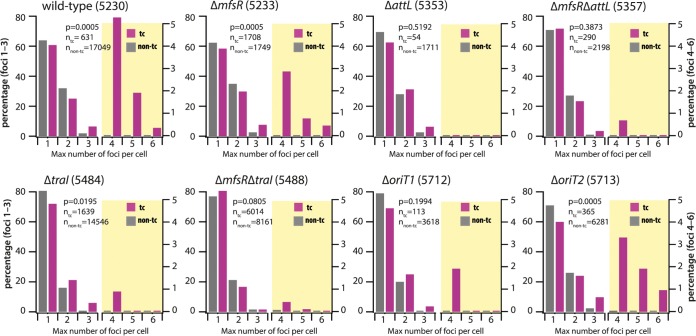
Distributions of LacI-CFP foci in transfer-competent (tc) and regular cells (non-tc) of P. putida carrying ICE*clc* (wild type) or ICE*clc* with different relevant mutations. Focus distributions are represented as the percentage of cells among the total of that category (*n*, as indicated in each diagram) with the indicated maximum observed number of foci during their entire lifetime. Different scales were used to show the percentages for focus numbers 1 to 3 (left scale axis) and for focus numbers 4 to 6 (right axis; diagram partly shaded in yellow). *P* values are the simulated *P* values across 2,000 repetitions of the comparisons of percent-normalized non-tc versus tc focus distributions in Fisher’s exact test. Data from strains 5230, 5233, 5357, and 5488 are in 5, 2, 2, and 2 biological replicates, respectively, with between 4 and 10 technical replicates. The other data are from single biological replicates with between 4 and 10 technical replicates.

In contrast, and although their overall number was much lower, ICE*clc* wild-type tc cells showed a very distinct focal pattern from non-tc cells at the same focus thresholding (Fig. 2c, tc cells; Fig. 3, wild type). During exponential growth, 17 to 56% of tc cells displayed two CFP foci (Fig. S3), and two foci were detected in cells even before the onset and after the end of population growth (Fig. 2c and Fig. S3). On average, 19% ± 13% of tc cells displayed three and up to six CFP foci compared to 2.0% ± 0.9% of non-tc cells (*P* = 0.0095, one-sided *t* test, *n* = 5; Fig. 2b; Fig. 3, wild type; and Fig. S3). The microcolony shown in Fig. 2b further illustrates the dynamic appearance of foci in tc cells. The distributions of maximum observed number of foci between tc and non-tc cells at any point during their lifetime were highly significantly different (Fig. 3, *P* = 0.0005 by Fisher’s exact test, *n* = 5 biological replicates). The consistently higher number of LacI-CFP foci suggested that the ICE had excised specifically in tc cells, as expected, and replicated in its excised form.

10.1128/mBio.01133-19.4 FIG S3 Biological replicates of focus formation in non-tc and tc cells in P. putida ICE*clc-lacO_ARRAY_*; *araC*,*lacI-cfp*; *P_inR_-echerry* (strain 5230). Proportional focus distributions over time of growth as in Fig. 2 in the main text, for all quantified foci, or per category of foci of ≥ 3. Numbers (n) indicate the total amount of cells observed for each category during the duration of the experiment. Indications on the side indicate the number of technical replicates grouped per stack plot. Download FIG S3, PDF file, 1.9 MB. Copyright © 2019 Delavat et al. 2019 Delavat et al. This content is distributed under the terms of the Creative Commons Attribution 4.0 International license.

**FIG 4:**
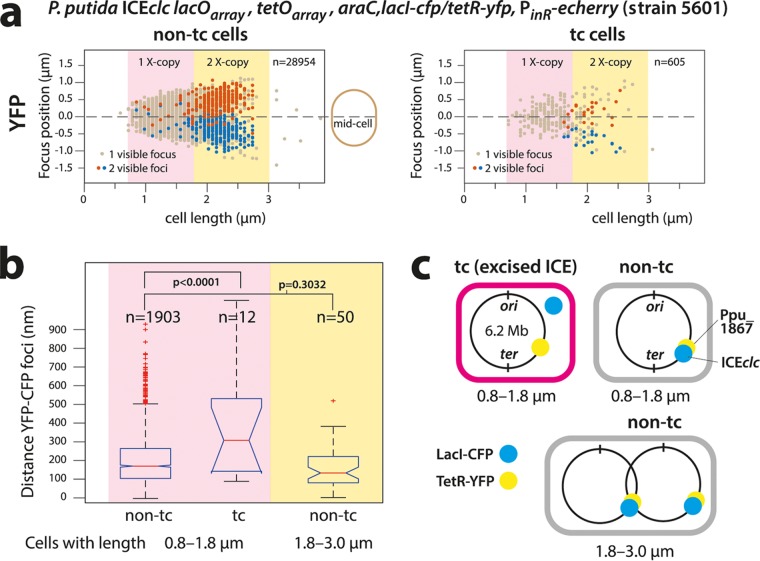
Excision of ICE*clc* in P. putida tc cells colabeled with LacI-CFP and TetR-YFP. (a) Focus positions plotted in micrometer distance along the midcell axis in individual non-tc or tc cells ordered as a function of cell length. Note detectable chromosome replication (double TetR-YFP foci) in cells of >1.8 μm (1X- or 2X-copy indicates 1 or 2 chromosomal copies, respectively). TetR-YFP foci in tc cells follow the general positional pattern of non-tc cells. Cells with focus pairs are arbitrarily colored red and blue, the red being the focus farthest away from the bottom cell pole. n, number of analyzed cells in each category. (b) Increased interfocal (TetR-YFP/LacI-CFP) distances in tc compared to non-tc cells (size range, 0.8 to 1.8 μm) with on average a single chromosome copy (*P* < 0.0001 in ANOVA followed by *post hoc* Tukey test) but not between TetR-YFP/LacI-CFP focus pairs in small and larger non-tc cells (*P* = 0.3032). Data (n, number of analyzed cells in that category) presented as notched box plots, with red lines showing the median and red plus signs showing the outliers. (c) Schematic positioning of the integrated ICE*clc lacO_ARRAY_* and *tetO_ARRAY_* on the P. putida chromosome. Note that it is unlikely that four pairs of foci would be visible as a result of renewed chromosome replication before daughter cell separation. Data in panels a and b grouped from 23 technical replicates.

To further confirm ICE*clc* excision in tc cells, we used a P. putida derivative (strain 5601) containing, in addition to the *lacO_ARRAY_* on the ICE itself, a *tetO_ARRAY_* integrated in the Pp_1867 locus 12 kb upstream of the position of ICE*clc attR* on the P. putida chromosome (Fig. 1d). We expected that upon ICE*clc* excision in tc cells, the distance between LacI-CFP and TetR-YFP foci would increase (Fig. 4). A diagram of TetR-YFP focus positions plotted as a function of the longitudinal cell axis size illustrates the ongoing chromosome replication in non-tc and tc cells (Fig. 4a). Cells with two observable YFP foci tended to have lengths of 1.8 μm upward up to a length of 2.5 to 3.0 μm, after which the two daughter cells separated (Fig. 4a). Longer non-tc cells displayed proportionally larger TetR-YFP interfocal distances, with positions symmetrical to the cell middle, indicative of segregating replicating chromosomes (Fig. 4a, red and blue dots). The majority of non-tc cells (90.4%) with a single YFP focus were smaller (0.8 to 1.8 μm), and the relative distance of that focus to the cell middle was less than 0.5 μm (Fig. 4a, light brown dots). Correspondingly, 89.1% of non-tc cells with a single CFP focus were sized between 0.8 and 1.8 μm. The average distance between CFP and YFP foci in non-tc cells with one chromosome copy (i.e., cell size range of 0.8 to 1.8 μm) or in cells with sizes between 1.8 and 3.0 μm with two visible foci of each (in the same replichore) was close and not significantly different (199 ± 126 nm versus 172 ± 111 nm, *P* = 0.3032 by ANOVA followed by *post hoc* Tukey test, Fig. 4b). This is indicative of integrated ICE*clc*, with closely juxtapositioned LacI-CFP and TetR-YFP binding sites (Fig. 4c).

Chromosome replication and segregation in tc cells followed the same trend as in non-tc cells (Fig. 4a, tc cells). In contrast, the distance between CFP and YFP focus positions in tc cells of 0.8 to 1.8 μm (single chromosome) was on average twice as large as in non-tc cells (385 ± 287 nm, *P* < 0.0001 by ANOVA, followed by *post hoc* Tukey test, Fig. 4b). This indicates that ICE*clc* and the nearby chromosome locus with the *tetO* array became physically separated, which is in agreement with the hypothesis of ICE*clc* being excised in tc cells.

### ICE-factor dependent replication of excised ICE.

In order to determine whether the observed multiple ICE copies in tc cells (three to six) were the result of a replicative process dependent on ICE*clc* factors, we compared CFP focus numbers in tc and non-tc cells in a variety of ICE mutant strains of P. putida.

The numbers of foci in a P. putida strain carrying an ICE in which the *attL* excision-recombination region was deleted (strain 5353, Table S1) did not exceed three (Fig. S2), and focus distributions among non-tc and tc cells were not significantly different (Fig. 3, Δ*attL*, *P* = 0.5192, Fisher’s exact test). As expected, focus distributions among tc cells were significantly different between wild-type and *ΔattL* mutant (Table 1, *P* = 0.0005). This is in agreement with previous findings by quantitative PCR that ICE*clc* cannot excise in this mutant (14). LacI-CFP foci in the Δ*attL* strain, therefore, solely indicated chromosomally integrated ICE copies.

**TABLE 1:** Comparison of LacI-CFP focus distributions among transfer-competent cells in P. putida ICE*clc* and relevant mutants

Comparison group	*P* value^*a*^
Wild type vs Δ*attL*	0.0005
Wild type vs Δ*traI*	0.0005
Wild type vs Δ*oriT1*	0.0020
Wild type vs Δ*oriT2*	0.3788
Δ*mfsR* vs Δ*mfsR*-Δ*traI*	0.0005
Δ*mfsR* vs Δ*mfsR*-Δ*attL*	0.0005
Δ*oriT1* vs Δ*oriT2*	0.0105

a Fisher’s exact test, *n* = 2,000 simulations, distributions scaled to the cell number of the first comparison group (e.g., wild type or Δ*mfsR*).

Focus numbers among tc cells in P. putida strain 5484 carrying ICE*clc* with a deletion in the *traI* gene (Fig. 1b) were significantly reduced compared to strain 5230 with wild-type ICE (Table 1, *P* = 0.0005) although still statistically different from that of non-tc cells (Fig. 3, Δ*traI*, *P* = 0.0195). Homologs of *traI* in other ICE systems have been implicated in replication of the excised ICE (17, 23). Focus numbers in tc cells of P. putida containing an ICE*clc* with a deletion in *oriT1*, one of the origins of transfer on which the TraI relaxase is acting (24), were lower than in wild-type tc cells (Table 1, *P* = 0.002) and not different than in non-tc cells (Fig. 3, Δ*oriT1*, *P* = 0.1994). In contrast, the focus distribution in tc cells of P. putida with a deletion in the alternative origin of transfer *oriT2* (24) was not different from that of wild type (Table 1, *P* = 0.3788), but it was different from that of Δ*oriT2* non-tc cells (*P* = 0.0005) and *ΔoriT1* tc cells (Table 1, *P* = 0.0105). This suggested that *oriT1* (but less so *oriT2*) is important for the replication of the excised ICE*clc* molecule.

In order to increase the number of observable tc cells, we further deployed a P. putida strain with an ICE*clc* deleted for the regulatory gene *mfsR* (Fig. 1b), equipped with the *lacO_ARRAY_* and the inducible *lacI-cfp* system (strain 5233). In this strain, the proportion of tc cells in stationary phase increased to 45.4% ± 6.3% (Fig. 2d) (25). P. putida ICE*clc-*Δ*mfsR* (strain 5233) cells showed some overt displays of multiple LacI-CFP foci in individual tc cells (Fig. 2e). This example is also illustrative of the dynamic movement of the various LacI-CFP foci over time in individual nondividing tc cells, suggesting some active mechanism for their redistribution (see Movie S1). Dividing non-tc cells of this ICE-hyperactive strain 5233 with deleted *mfsR* still mostly displayed one or two LacI-CFP foci (Fig. 3, Δ*mfsR*). In contrast, tc cells carried significantly higher proportions of 3, 4, and up to 6 foci than non-tc cells (*P* = 0.0005, Fisher’s exact test).

**FIG 5:**
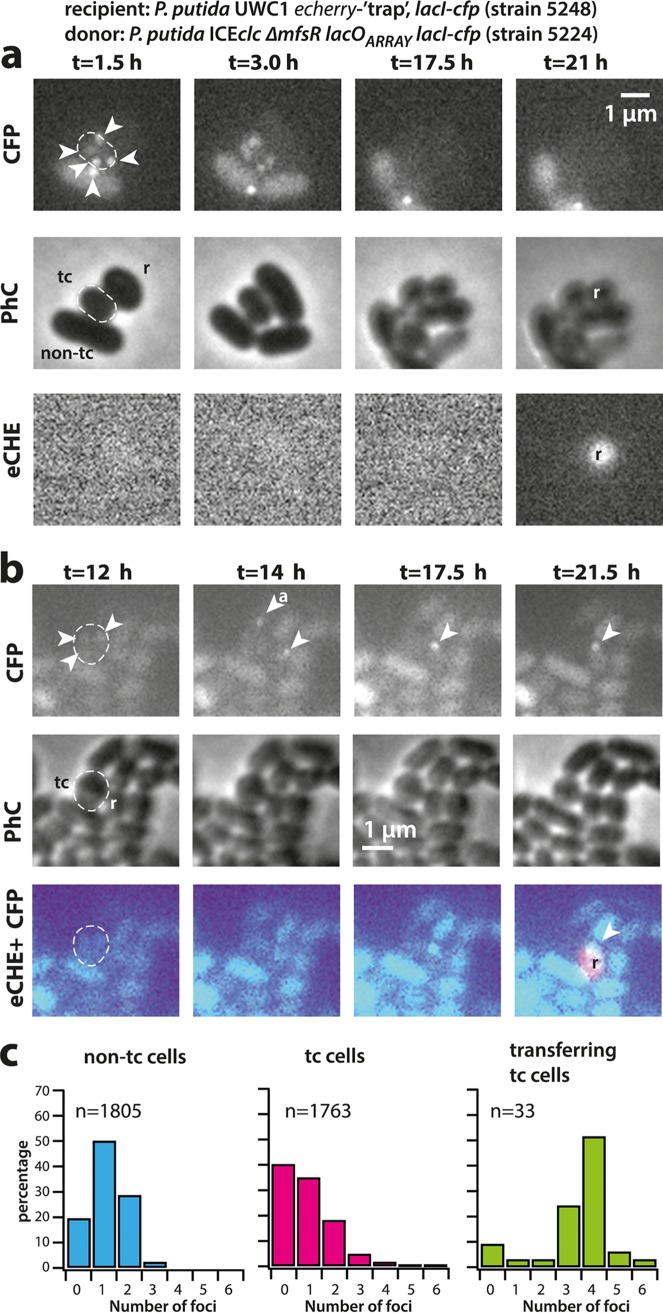
ICE transfer is favored from tc cells with higher copy number of excised ICE*clc*. (a and b) Examples of ICE*clc* transfer from tc donor cells with excised and replicated ICE (note the 3 to 5 visible LacI-CFP foci in donor cells, dashed outlines) to neighboring ICE-free recipient cells with the conditional trap (r) and appearance of eCherry fluorescence (eCHE) as a result of ICE integration (*t* = 21 h in panel a and *t* = 21.5 h in panel b). Note how a LacI-CFP focus appears in each of two recipient cells in panel b (*t* = 14 h), only one of which is integrated, whereas the other may be aborted (“a”). (c) Observed focus distributions (maximum per cell lifetime, in percentage of total) in tc donor cells with successful ICE*clc* transfer to recipient, compared to the focus distributions of all non-tc and tc cells of the same strain in the absence of recipient. Data in panel c are from two (non-tc and tc) and four (transfer) independent biological replicates. Each biological replicate contains 3 technical replicates (i.e., different patches).

10.1128/mBio.01133-19.8 MOVIE S1 ICE*clc* transfer between P. putida ICE*clc* Δ*mfsR lacO_ARRAY_ lacI-cfp* (strain 5224) as donor and P. putida UWC1 *echerry*-‘trap’, *lacI-cfp* (strain 5248) as recipient. Different area as in Fig. 5a and b in the main text, enlarged to the complete microscope view. Time steps, 30 min. Shown is an overlay of CFP (cyan) + eCHE (magenta). Note the dynamic movement of foci in donor cells (example, still image—region a) and appearance of transconjugants in which ICE*clc* is stably integrated by their continuous eCHE color (region b example). Note further how some transconjugants briefly appear before lysing and disappearing (area near region c). Download Movie S1, AVI file, 8.5 MB. Copyright © 2019 Delavat et al. 2019 Delavat et al. This content is distributed under the terms of the Creative Commons Attribution 4.0 International license.

Compared to ICE*clc*-Δ*mfsR*, the additional Δ*traI* deletion diminished focus numbers in tc cells (Table 1, *P* = 0.0005; Fig. 3), but focus distributions among non-tc and tc cells were not different (Fig. 3, Δ*mfsR*Δ*traI*, *P* = 0.0805). Also in the P. putida strain carrying ICE*clc*-Δ*mfsR*-Δ*attL*, the focus distributions between non-tc and tc cells were indistinguishable (Fig. 3, Δ*mfsR* Δ*attL*, *P* = 0.3873), whereas tc cell focus numbers were lower in comparison to P. putida ICE*clc-*Δ*mfsR* (Table 1, *P* = 0.0005). Collectively, these results indicated, therefore, that the higher LacI-CFP focus numbers in tc cells of wild-type and Δ*mfsR* strains are indeed the result of a replicative process that involves the TraI relaxase and *oriT1*, but less so *oriT2*. Compared across all strains, the proportions of cells without any visible foci were higher in tc than in non-tc cells (*P* = 2.2 × 10^−7^, single-sided *t* test, Fig. S4), but no effect of ICE*clc* gene deletions was detected (Fig. S4, ANOVA, *P* = 0.5057).

10.1128/mBio.01133-19.5 FIG S4 Proportions of cells without any detectable foci in non-tc and tc cells of P. putida ICE*clc-lacO_ARRAY_*; *araC*,*lacI-cfp*; *P_inR_-echerry* with mutations in critical ICE excision or replication functions. Error bars indicate calculated standard deviations from the mean of biological replicates. *P* value of testing the proportions between non-tc and tc cells across all strains (single-sided *t* test, hypothesis that tc cells have higher proportions of cells with any detected foci). Download FIG S4, PDF file, 0.7 MB. Copyright © 2019 Delavat et al. 2019 Delavat et al. This content is distributed under the terms of the Creative Commons Attribution 4.0 International license.

### Cells with more excised ICE*clc* copies transfer more frequently.

To test whether higher ICE copy numbers in tc cells would increase the success of conjugation, we mixed donors of P. putida ICE*clc-ΔmfsR* tagged with *lacO_ARRAY_* and LacI-CFP with a conditional fluorescent P. putida recipient strain as bait. The recipient strain (strain 5248, Table S1) fuses a promoterless *echerry* gene downstream of the *attB* recombination site (14) and additionally expresses LacI-CFP. Integration of ICE*clc* into the conditional trap results in placement of the constitutive outward-facing P_circ_ promoter (Fig. 1b) directly upstream of *echerry*. Even though this strain captures only ∼20% of all integration events (the others going into alternative *attB* sites on the chromosome formed by genes for tRNA-glycine [26]), one can quantify the numbers of foci in donor cells appearing in contact with eCherry-forming recipients and compare their LacI-CFP focus distribution to that observed in all tc donor cells without recipient. Figure 5 shows two distinct examples of such transfer events. In Fig. 5a, the tc donor cell displays 4 to 5 LacI-CFP foci (visible after 1.5 to 3 h), leading to an eCherry-producing transconjugant visible at *t* = 21 h. In Fig. 5b one can see how the incoming ICE*clc* is bound by the recipient’s LacI-CFP (shown at *t* = 14 h in two cells neighboring the tc donor cell). One of those disappears over time, possibly as a result of aborted replication and lack of integration (cell labeled with “a” in Fig. 5b; *t* = 14 h). The other LacI-CFP remains and eventually leads to a recipient producing eCherry (at *t* = 21.5 h), indicative of its proper integration into the conditional trap (Fig. 5b; the full time series of both events is shown in Fig. S5). On average, we found a time span of 3.5 to 10 h between the appearance of a LacI-CFP recipient focus (indicative of ICE transfer to the recipient) and detectable eCherry expression (indicative of ICE integration in the recipient’s genome). Across 33 detected transfer events with ICE*clc* integrated in the recipient’s conditional trap, the identified tc cell donors displayed more LacI-CFP foci than expected from the focus distribution seen for tc cells in general (Fig. 5c, *P* value = 0.0005 in Fisher’s exact test comparing focus distributions). This is thus a strong indication that donors with multiple ICE copies preferentially contribute to ICE transfer.

10.1128/mBio.01133-19.6 FIG S5 Full relevant time steps in ICE*clc* transfer between P. putida ICE*clc* Δ*mfsR lacO_ARRAY_ lacI-cfp* (strain 5224) as donor and P. putida UWC1 *echerry*-‘trap’, *lacI-cfp* (strain 5248) as recipient. (Expanded data from Fig. 5a and b in the main text.) Time steps, 30 min. PhC, phase contrast; CFP, cyan fluorescent protein; eCHE, eCherry fluorescence. Overlay in panel a, PhC + CFP (cyan) + eCHE (magenta). Overlay in panel b, CFP (cyan) + eCHE (magenta). Download FIG S5, PDF file, 1.3 MB. Copyright © 2019 Delavat et al. 2019 Delavat et al. This content is distributed under the terms of the Creative Commons Attribution 4.0 International license.

## DISCUSSION

It is increasingly recognized that selfish DNA elements mediating horizontal gene transfer have lifestyles of their own, which are subject to adaptation and selection (3). This is not only interesting as a biological or molecular curiosity but crucial to understand, given the role of such elements in promoting antibiotic resistance and xenometabolism in microbial communities (27). Eventually, this adaptation and selection may result in some elements being more successful in distributing these genes than others, and some conditions may preferentially select for successful DNA-transferring elements. ICEs may be particularly relevant for this question as they have evolved exquisite regulatory systems to control their lifestyle, both in their vertical modes of coreplication with the bacterial chromosome and in the switch to horizontal transfer (1–3). Although several different ICE models have contributed to the molecular understanding of ICE functioning; in particular, studies on the ICE*clc* in *P. knackmussii* and P. putida have helped to elucidate the context of cellular differentiation and ecological fitness (12). As a result of spontaneous recent gene integration (28), wild-type ICE*clc* is transferring at a sufficiently high rate (1 per 100) that single-cell studies can be conducted, whereas most other wild-type ICEs transfer at rates of 1 per 10^5^ or less (estimated in reference 3). Single-cell studies revealed that ICE*clc* transfers only from differentiated tc cells arising under stationary-phase conditions (14, 21). The fitness strategy of the ICE is thus based on two pillars: vertical descent through coreplication with the chromosome and horizontal transfer via specialized tc cells. Since cell division is perturbed in tc cells as a result of the ICE*clc* activation process, too-high transfer rates would compromise fitness of the host-ICE population of cells (14). Too-low transfer would not be in the fitness interest of the ICE, so that a (dynamic) equilibrium arises that balances fitness loss in tc cells with ICE fitness gain from horizontal transfer and vertical descent (14).

Interestingly, and this has been rarely recognized, the transient existence of tc cells necessitates ICE*clc* to transfer as optimally as possible in order to maximize horizontal fitness. The adaptations for this process can be seen only at individual cell level. As an example, we show here that a transient phase of replication of excised ICE molecules in tc cells favors more effective transfer (Fig. 5c), which is a selectable feature increasing the ICE horizontal fitness. Although we could not capture all possible ICE transfers, because many of them were invisible to the conditional trap, the majority of imaged transferring tc donor cells displayed more LacI-CFP foci than expected by chance from the distribution of LacI-CFP foci seen among all tc cells (Fig. 5c, *P* = 0.0005). This does not exclude the possibility that cells with fewer LacI-CFP foci (and consequently, lower numbers of excised ICE molecules) transfer at all but indicates that tc cells with higher numbers of ICE replicons have a higher chance of transferring the ICE. Mechanistically, this may occur through independent ICE transfer events at multiple positions around the cell or through enhanced delivery rates of multiple single-stranded ICE DNA at a single conjugative pore. Visual occurrence of quasisimultaneous multiple transfers from single donor to different neighboring recipient cells (as in Fig. 5b and in reference 14) would favor the hypothesis of existence of multiple transfer pores, although these have so far not been seen.

Single-molecule single-cell studies for ICE behavior, as shown here, have important advantages over the use of population-based qPCR, but inherently have other pitfalls, which we tried to control for as well as possible. The LacI-CFP system is not perfect, and as a consequence of image focusing or cell-cell variability of induction by arabinose, ICE*clc* “foci” may be missed in individual cells. Thresholding between spurious and real ICE*clc*-originated fluorescence foci inevitably results in some 10% of cells without detectable foci (see Fig. S2 and S4 in the supplemental material), whereas such strains show no evidence for losing the ICE. At the same conservative focus threshold settings, however, the high number of foci seen specifically in tc compared to non-tc cells of wild-type and Δ*mfsR* mutant strains is experimentally robust and statistically significant (Fig. 3) and is consistent with the concept of replicating excised ICE*clc* in tc cells. The presence of two foci is explained by ongoing chromosome replication in dividing but not yet separated cells. Given the integration position of ICE*clc* in the P. putida chromosome close to the *ter* region (Fig. 4c), however, it is unlikely that renewed chromosome replication at the *ori* in dividing cells could produce 4 CFP foci. Although small percentages of three CFP foci in non-tc cells or in tc cells of the *attL* mutant (Fig. 3) may be attributed to insufficiently conservative focus thresholding (Fig. S2), they might also be the result of specific replication of integrated ICE*clc*, as was deduced and postulated for ICE*Bs1* in Bacillus subtilis (16, 29, 30), and more recently seen for certain prophages (31). Colabeling experiments showed that on average the TetR-YFP and LacI-CFP foci separate in tc cells but not in non-tc cells. This indicates that the ICE molecule physically disengaged from its nearby chromosomal location and consequently must have been excised in tc cells. Most likely, therefore, even two CFP foci in small tc cells (<1.8 μm) reflect excised ICE*clc*, and any number of foci equal to or larger than three in tc cells indicates replication of excised ICE*clc*. This conclusion is consistent with the striking difference in focus distributions in mutants without the *attL* recombination site, in which the ICE is chromosomally locked (Fig. 3). Further consistent with population-based studies on other ICEs in B. subtilis and V. cholerae (17, 23), a *traI* relaxase seems to be responsible for the replication of the ICE extrachromosomal form, and both *traI* and *oriT1* but not *oriT2* deletions diminished the appearance of tc cells with more than three CFP foci (Fig. 3). Of note is that a very small proportion of tc cells in such mutants still displayed 4 foci (Fig. 3), which may point to further alternative replication mechanisms. In summary, our data thus consistently show that focus numbers of ≥3 in tc cells represent replicated excised ICE*clc* molecules.

What role(s) may excision replication play in the case of ICE*clc*? We noticed that the proportion of cells without any visible CFP foci in general was higher among tc than non-tc cells (Fig. S4), suggesting that part of tc daughter cells indeed lose ICE*clc*. This would be consistent with a previous inference of ∼20% potential ICE*clc* loss in tc cells (14). In contrast, our data did not show a statistically significant increase of the proportion of tc cells without any foci in strains lacking *traI* or *oriT1/2*, nor a decrease in *attL* mutants (Fig. S4). We acknowledge therefore that ICE*clc* has a risk to be lost in dividing tc cells, but we cannot conclude that this risk increases when excised ICE replication is impaired. Therefore, we cannot confirm that replication would contribute to ICE maintenance in dividing tc cells, as has been concluded from population-based studies on ICE in B. subtilis and V. cholerae (16–18). A maintenance mechanism in tc cells for excised ICE*clc* seems less ecologically relevant, given their death and disappearance in exponentially growing cultures (12, 14). Rather, our data indicate that a higher number of excised ICE*clc* molecules directly translates into a higher rate or success of transfer and, therefore, gain of horizontal fitness.

## MATERIALS AND METHODS

### Strains and culture conditions.

Escherichia coli strains used for plasmid cloning were routinely cultured at 37°C in LB medium, while P. putida strains were grown at 30°C in LB or in 21C minimal medium (MM) (32) supplemented with 5 mM 3-chlorobenzoate (3-CBA) or 10 mM sodium succinate. Antibiotics were used at the indicated concentrations, if necessary: ampicillin (Amp), 100 μg ml^−1^; gentamicin (Gm), 20 μg ml^−1^; kanamycin (Km), 50 to 100 μg ml^−1^. Strains used in this study are listed in Table S1 in the supplemental material.

ICE*clc* maintenance in P. putida cultures was verified by replica plating. A single colony grown for 16 h on a freshly streaked LB-Km plate from the −80°C stock was inoculated in 10 ml liquid LB with the required antibiotics for marker selection. Aliquots of 100 μl were transferred to 20 ml of MM with succinate or with 3-CBA (in the absence of antibiotics) and grown to stationary phase (96 h) and from there serially diluted and plated on MM agar plates with succinate (without antibiotics). One hundred colonies from each (LB to MM succinate or LB to MM 3-CBA) were then replica plated on MM agar with succinate and MM agar with 3-CBA. For none of the strains (5230, 5233, 5353, 5357, 5484, 5488, 5712, and 5713, Table S1) was any of the succinate-grown colonies unable to grow on MM with 3-CBA. Hence, ICE*clc* was maintained in the absence of selection in more than 99.5% of cells.

### Strain constructions and DNA techniques.

DNA manipulations and molecular techniques were performed according to standard procedures (33) and recommendations by the reagent suppliers. Targeted chromosomal deletions and insertions in P. putida were created by recombination with nonreplicating plasmid constructs and counterselection techniques as previously described (20, 34). Recombination was facilitated by including regions up- and downstream of the targeted positions with sizes of approximately 0.7 kb.

To visualize and quantify ICE*clc*-containing DNA molecules in individual cells, we deployed the technique of *in vivo* binding of fluorescently labeled LacI or TetR to multiple tandem copies of their cognate binding sites (19). A DNA fragment containing two times 120 *lacO* copies (*lacO_ARRAY_*), each interspaced by 10 random bp and with a Km resistance gene in the middle (19), was inserted within the *amnB* gene of ICE*clc* (8) in P. putida (Fig. 1b). The *amnB* gene is part of a metabolic pathway involved in the degradation of 2-aminophenol and nonessential for the conjugative transfer of ICE*clc* (8). The corresponding fragment was recovered from pLAU43 (19) using digestion with BamHI and SalI and cloned into the *Pseudomonas* recombination vector pEMG (34) (accession number JF965437), flanked by two 0.7-kb recombination fragments surrounding *amnB*. Similarly, a *tetO* array consisting of two times 120 *tetO* binding sites separated by a Gm resistance gene was extracted from pLAU44 (19) using NheI and XbaI, cloned into pEMG, and flanked with two fragments for recombination next to the gene Pp_1867, which is located 12 kb upstream of the insertion site of ICE*clc* in the genome of P. putida (accession number NC_002947.4). Proper recombination and marker insertion were verified by PCR amplification and sequencing.

The *araC*, *lacI-cfp* fragment used for arabinose-inducible expression of LacI-CFP under the control of AraC was amplified from pLAU53 (19), verified by sequencing, and cloned in a mini-Tn*7* delivery plasmid (35) (accession number AY599231) using SpeI and HindIII. The *araC*, *lacI-cfp*, *tetR-yfp* fragment used for ectopic expression of both LacI-CFP and TetR-YFP was retrieved from pLAU53 using digestion with SgrAI and HindIII and inserted into the mini-Tn*7* delivery plasmid at XmaI and HindIII positions. The resulting plasmids were cotransformed with the Tn*7*-expressing helper plasmid pUX-BF13 (36) into the different P. putida strains (Table S1). After selection of transformants for the respective antibiotic resistance markers expressed by the mini-Tn*7* cassette, its proper site-specific insertion at the *glmS* site was verified by PCR amplification.

To differentiate non-tc and tc cells, we used the ICE tc-cell specific P_inR_ promoter (13, 20), which was fused to a promoterless *echerry* gene in a transcriptionally shielded mini-Tn*5* transposon and integrated in single copy on the P. putida chromosome using a mini-Tn*5* delivery vector, as previously described (14). Three independent mini-Tn*5* insertions were kept for each derivative strain.

### Epifluorescence microscopy.

Fluorescent protein expression in individual cells was examined by epifluorescence microscopy on a Nikon Eclipse Ti-E inverted microscope, equipped with a perfect focus system (PFS), pE-100 CoolLED, and a Plan Apo l 100× 1.45 oil objective (Nikon), installed in a controlled-temperature room (22°C).

Cell growth and tc cell development were followed in 50-h-long time-lapse experiments, with cells seeded on round (ca. 1-cm diameter, 1 mm thick) 1% agarose disks placed inside closed sterilized metal microscope chambers (12, 14, 37). Surfaces were inoculated with late-stationary-phase (96-h) precultures, to ensure the presence of tc cells at the beginning of the experiment. Precultures were prepared by transferring 100 μl of an overnight-grown culture on LB with antibiotics to maintain selection of the chromosomal markers into an Erlenmeyer flask containing 20 ml of MM with 5 mM 3-CBA (without antibiotics). This culture was incubated for 96 h at 30°C with 200-rpm rotary shaking (cells reach stationary phase after 24 h). If relevant, at this point l-arabinose was added to the culture at a final concentration of 100 mg liter^−1^ to induce expression of *lacI-cfp* from P_BAD_. After a 90-min incubation, 1 ml of the culture was centrifuged for 2 min at 18,000 × *g* to collect the cells, which were resuspended in 10 ml MM without added carbon substrate. Six microliters of this washed preculture was then spread per agarose disk, which further contained 0.1 mM 3-CBA in MM and 10 mg liter^−1^ l-arabinose to maintain induction from P_BAD_.

For observation of chromosome replication in dividing P. putida cells with both LacI-CFP and TetR-YFP labeling (strain 5601), we imaged cells directly (i.e., without time-lapse) from a liquid culture in MM with 5 mM 3-CBA and 10 mg liter^−1^ l-arabinose incubated for 4 h at 30°C. For imaging, cells were concentrated and resuspended as described above and spread on 1% agarose surface on microscope slides. This culture was prepared by 10-fold dilution from a preculture in MM with 5 mM 3-CBA that had been grown to stationary phase for 48 h (to ensure tc cell development), after which 100 mg liter^−1^ l-arabinose had been added for 90 min to express LacI-CFP and TetR-YFP. An incubation of 4 h is sufficient to revive the cells from stationary phase and resume cell division in tc and non-tc cells (note that any tc cells lysing within this period will be lost from the analysis).

In the case of ICE*clc* time-lapse transfer experiments, donor cells were prepared as described above. The P. putida recipient strain with the conditional eCherry-fluorescent ICE-integration trap and ectopically expressing LacI-CFP (strain 5248) was grown with 10 mM succinate for 24 h and incubated with 100 mg liter^−1^ l-arabinose for 90 min. Donor and recipient cells were washed as described above, resuspended in MM without carbon source, mixed in a 1:2 (vol/vol) ratio, respectively, and seeded on a 1% agarose disk surface with 0.1 mM 3-CBA and 10 mg liter^−1^ l-arabinose as previously done for donor cells alone.

Seeded agarose disks were turned upside down, cells facing the lower coverslip, and enclosed in an autoclaved microscopy chamber (Perfusion Chamber; H. Saur Laborbedarf, Germany). Assembled chambers contained four simultaneous patches, one of which remained noninoculated and served to pause the microscope objective between imagings and avoid light-induced stress on the cells. Chambers were adapted for 1 h to the temperature (22°C) and humidity of the microscope room, before starting the time-lapse experiment. Images were recorded at a light intensity of 10% (solar light engine, LED power 4%) and an ORCA-Flash 4.0 camera (Hamamatsu). Exposure times for phase-contrast images were 50 or 100 ms, for eCherry fluorescence were 20 or 50 ms, for CFP fluorescence were 200 or 250 ms, and for YFP fluorescence were 250 ms. Four positions on each disk were programmed in MicroManager (version 1.4.22) and were imaged every 30 min during 50 h.

As biological replicates, we define independently seeded agarose disks from separate precultures, either within the same microscope chamber started on the same day or inoculated at different times. As technical replicates, we define cells imaged at different positions on the same agarose disk.

### Image analysis.

Fluorescence values of single cells obtained from snapshot microscopy experiments were extracted using an in-house Matlab script as described previously (14), and subpopulations were quantified from quantile-quantile plotting (21). tc cells were categorized on the basis of eCherry fluorescence expressed from the single-copy P_inR_ promoter in quantile-quantile analysis, which scores the deviation of the observed distribution of eCherry fluorescence among individual cells to the expected normal distribution assuming a single population (12, 21).

Individual cells in time-lapse image series (up to 100 frames) were segmented using SuperSegger (22), and both cellular fluorescence and the fluorescence intensities, scores, and positions of up to 9 foci in individual cells were extracted. Optimized segmentation constants for P. putida were derived from the “training your own constants” subprocedure in SuperSegger. Extracted data were then analyzed with a homemade Matlab script (Text S1) (i) to identify tc and non-tc cells; (ii) to derive the genealogy of all cells and link them within growing microcolonies (cell ID, frame of birth, frame of death, mother and daughter IDs); and (iii) to count the position, number, normalized fluorescence intensity, and scores of individual cell foci over time. tc cells were identified in the first image frame on the basis of quantile-quantile plotting of eCherry fluorescence values as the subpopulation with the highest eCherry fluorescence, whereas the largest subpopulation with the lower average eCherry fluorescence was considered to contain non-tc cells. Mother cells forming microcolonies of less than 8 cells were further excluded from the group of non-tc cells, since they may consist of tc cells with low eCherry starting values. This procedure was justified based on previous observations of poor regrowth of tc cells (12). Foci from segmented cells were thresholded on score and intensity, from values of 1 to 7. In most cases, score and intensity values of 6 satisfied the criteria of (i) no detected foci in P. putida strains without *lac_ARRAY_* or LacI-CFP, (ii) minimal number of cells with more than two foci in *attL* mutant strains (Fig. S2), and (iii) a maximum number of foci of 8. Individual cells with more than 4 foci were examined manually using the superSeggerViewerGUI mode of SuperSegger, and incorrectly segmented cells were removed from the final analysis. Focus distributions among the groups of tc and non-tc cells were normalized to within-group percentages and compared using Fisher’s exact test, given the absence of an *a priori* distribution function. Focus distributions among tc cells of the wild type and mutants were scaled to the total number of the tc cells of the first comparison group (i.e., wild type or Δ*mfsR*) before comparison in Fisher’s exact test (Table 1).

10.1128/mBio.01133-19.1 TEXT S1 Matlab code used for image analysis. Download Text S1, TXT file, 0.01 MB. Copyright © 2019 Delavat et al. 2019 Delavat et al. This content is distributed under the terms of the Creative Commons Attribution 4.0 International license.
