# Supplementary material for: Transient Replication in Specialized Cells Favors Transfer of an Integrative and Conjugative Element
Source: mBio. 2019 Jun 11;10(3):e01133-19. doi: 10.1128/mBio.01133-19 (PMC6561031; doi:10.1128/mBio.01133-19)
Supplement: FIG S1 [file mBio.01133-19-sf001.pdf]

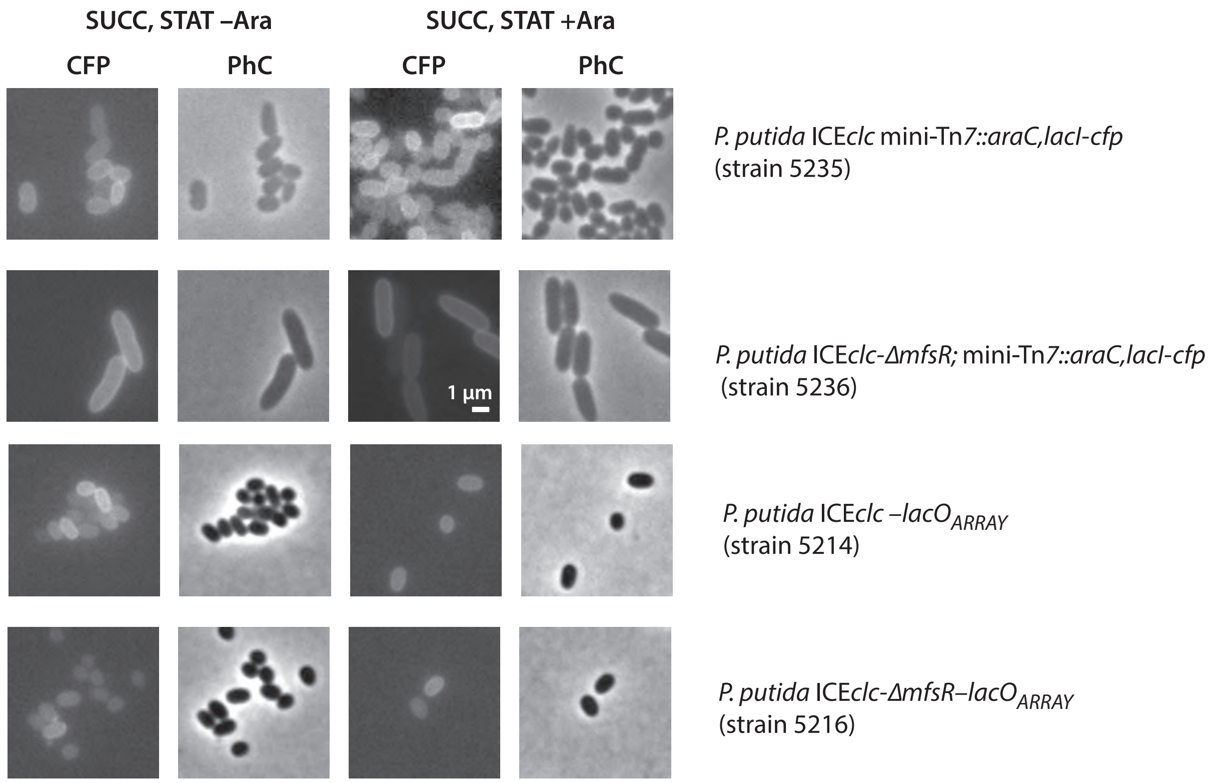

Supplementary Figure S1 |

Specificity of LacI-CFP fusion formation in *P. putida* cells.

P. putida with ICE*clc* and expressing *lacI-cfp* but without *lacO*$_{ARRAY}$, or with ICE*clc* and integrated *lacO*$_{ARRAY}$ but without ectopic insertion of *lacI-cfp* does not produce visible CFP foci in stationary phase (STAT), neither in absence of or in presence of added L-arabinose (Ara). Cells imaged 24 h after inoculation on minimal medium with 10 mM succinate (SUCC) as sole carbon source in phase contrast (PhC) or in epifluorescence with CFP filter (CFP). Note that the slight fluorescence 'ring'-shapes even in absence of LacI-CFP (as in strain 5214 and 5216) is due to siderophore production.
