# Supplementary material for: Transient Replication in Specialized Cells Favors Transfer of an Integrative and Conjugative Element
Source: mBio. 2019 Jun 11;10(3):e01133-19. doi: 10.1128/mBio.01133-19 (PMC6561031; doi:10.1128/mBio.01133-19)
Supplement: TABLE S1 [file mBio.01133-19-st001.pdf]

Table S1: Strains used in this study.

Collection number	Strain	Characteristics[a]	Reference or source
	Escherichia coli		
3044	DH5α λpir	*sup E44, ΔlacU169 (ΦlacZΔM15), recA1, endA1, hsdR17, thi-1, gyrA96, relA1, λpir* phage lysogen	V. de Lorenzo
	Pseudomonas putida		
1291	UWC1	Plasmid-free derivative of *P. putida* KT2440, Rif^R	[1]
2737	UWC1-*clc*5	Derivative of strain 1291 with one ICE*clc* copy integrated into $tRNA^{gly}$-5	[2]
4322	UWC1-*clc*5, Δ*mfsR*	Derivative of strain 2737 with an internal deletion in *mfsR*	[3]
5001	UWC1 *attB-echerry*	Derivative of strain 1291 (ICE*clc* free) with the mini-Tn*5 attB-echerry* conditional fluorescent trap. Used as mating recipient.	[4]
5214	UWC1-*clc*5, $lacO_{ARRAY}$	Derivative of strain 2737 with the *lacO* array inserted within *amnB* on ICE*clc*	This study
5216	UWC1-*clc*5, Δ*mfsR*, $lacO_{ARRAY}$	Derivative of strain 4322 with the *lacO* array inserted within *amnB* on ICE*clc*	This study
5222	UWC1-*clc*5, $lacO_{ARRAY}$, Tn7 *araC;lacI-cfp*	Derivative of strain 5214 with a mini-Tn7 *araC*-P_{BAD}-*lacI-cfp* insertion	This study
5224	UWC1-*clc*5, Δ*mfsR*, $lacO_{ARRAY}$, Tn7 *araC;lacI-cfp*	Derivative of strain 5216 with a mini-Tn7 *araC*-P_{BAD}-*lacI-cfp* insertion	This study
5228-5230	UWC1-*clc*5, $lacO_{ARRAY}$, Tn7 *araC; lacI-cfp,* Tn*5* P_{inR}-*echerry*	Derivatives of 5222 with a mini-Tn*5* P_{inR}-*echerry* insertion	This study
5231-5233	UWC1-*clc*5, Δ*mfsR*, $lacO_{ARRAY}$, Tn*7 araC;lacI-cfp,* Tn*5* P_{inR}-*echerry*	Derivatives of 5224 with a mini-Tn*5* P_{inR}-*echerry* insertion	This study
5235	UWC1-*clc5* Tn*7 araC;lacI-cfp*	Derivative of strain 2737 with the mini-Tn*7 araC*-P_{BAD}-*lacI-cfp* insertion	This study
5236	UWC1-*clc5* Δ*mfsR*, Tn*7 araC;lacI-cfp*	Derivative of strain 4322 with the mini-Tn*7 araC*-P_{BAD}-*lacI-cfp* insertion	This study
5248	UWC1 *attB-echerry,* Tn*7 araC;lacI-cfp*	Derivative of strain 5001 with mini-Tn*7 araC*-P_{BAD}-*lacI-cfp* insertion. ICE*clc* free. Used as mating recipient.	This study
5262	UWC1-*clc*5, Δ*attL,* $lacO_{ARRAY}$, Tn*7 araC;lacI-cfp*	Derivative of strain 5222 with a deletion of the *attL* site	This study
5345	UWC1-*clc*5, Δ*mfsR,* Δ*attL,* $lacO_{ARRAY}$	Derivative of strain 5216 with a deletion of the *attL* site	This study
5349	UWC1-*clc*5, Pp_1867-$lacO_{ARRAY}$	Derivative of strain 2737 with the *lacO* array inserted close to Pp_1867 (12 kb upstream of ICE*clc)*	This study
5351	UWC1-*clc*5, Δ*mfsR,* Δ*attL,* $lacO_{ARRAY}$, Tn*7 araC;lacI-cfp*	Derivative of strain 5345 with the mini-Tn*7 araC*-P_{BAD}-*lacI-cfp* insertion	This study
5352-5355	UWC1-*clc*5, Δ*attL,* $lacO_{ARRAY}$, Tn*7 araC;lacI-cfp,* Tn*5* P_{inR}-*echerry*	Derivatives of strain 5262 with a mini-Tn*5* P_{inR}-*echerry* insertion	This study

5357-5359	UWC1-*clc*5, Δ*mfsR,* Δ*attL,* $lacO_{ARRAY}$, Tn*7 araC;lacI-cfp,* Tn*5* P_{inR}-*echerry*	Derivatives of strain 5351 with a mini-Tn*5* P_{inR}-*echerry* insertion	This study
5361	UWC1-*clc*5, Pp_1867-$lacO_{ARRAY}$, Tn*7 araC;lacI-cfp*	Derivative of strain 5349 with the mini-Tn*7 araC*-P_{BAD}-*lacI-cfp* insertion	This study
5451	UWC1-*clc*5, Δ*traI, lacO*$_{ARRAY}$	Derivative of strain 5214 with a deletion in the relaxase gene (*orf50240*)	This study
5466	UWC1-*clc*5, Δ*mfsR,* Δ*traI,* $lacO_{ARRAY}$	Derivative of strain 5216 with a deletion in the relaxase gene (*orf50240*)	
5468	UWC1-*clc*5, Δ*traI*, $lacO_{ARRAY}$, Tn*7 araC;lacI-cfp*	Derivative of strain 5451 with the mini-Tn*7 araC*-P_{BAD}-*lacI-cfp* insertion	This study
5469	UWC1-*clc*5, Δ*mfsR*, Δ*traI*, $lacO_{ARRAY}$, Tn*7 araC;lacI-cfp*	Derivative of strain 5466 with the mini-Tn*7 araC*-P_{BAD}-*lacI-cfp* insertion	This study
5484-5487	UWC1-*clc*5, Δ*traI,* $lacO_{ARRAY}$, Tn*7 araC;lacI-cfp,* Tn*5* P_{inR}-*echerry*	Derivatives of strain 5468 with a mini-Tn*5* P_{inR}-*echerry* insertion	This study
5488-5491	UWC1-*clc*5, Δ*mfsR,* Δ*traI,* $lacO_{ARRAY}$, Tn*7 araC;lacI-cfp,* Tn*5* P_{inR}-*echerry*	Derivatives of strain 5469 with a mini-Tn*5* P_{inR}-*echerry* insertion	This study
5588	UWC1-*clc*5, Pp_1867-$tetO_{ARRAY}$, $lacO_{ARRAY}$	Derivative of strain 5214 with the insertion of a *tetO* array close to P1867 (12 kb upstream of ICE*clc)*	This study
5589	UWC1-*clc*5, Pp_1867-$tetO_{ARRAY}$, Δ*mfsR,* $lacO_{ARRAY}$	Derivative of strain 5216 with the insertion of a *tetO* array close to P1867 (12 kb upstream of ICE*clc)*	This study
5591	UWC1-*clc*5, P1867-$tetO_{ARRAY}$, $lacO_{ARRAY}$, Tn*7 araC;lacI-cfp /tetR-yfp*	Derivative of strain 5588 with the mini-Tn*7 araC*-P_{BAD}-*lacI-cfp; tetR-yfp* insertion	This study
5592	UWC1-*clc*5, P1867-$tetO_{ARRAY}$, Δ*mfsR,* $lacO_{ARRAY}$, Tn*7 araC;lacI-cfp /tetR-yfp*	Derivative of strain 5589 with the mini-Tn*7 araC*-P_{BAD}-*lacI-cfp; tetR-yfp* insertion	This study
5597	UWC1-*clc*5, Δ*oriT1,* $lacO_{ARRAY}$	Derivative of strain 5214 with a deletion in *oriT1*	This study
5598	UWC1-*clc*5, Δ*oriT2,* $lacO_{ARRAY}$	Derivative of strain 5214 with a deletion in *oriT2*	This study
5599	UWC1-*clc*5, Δ*mfsR,* Δ*oriT1,* $lacO_{ARRAY}$	Derivative of strain 5216 with a deletion in *oriT1*	This study
5601-5603	UWC1-*clc*5, P1867-$tetO_{ARRAY}$, $lacO_{ARRAY}$, Tn*7 araC;lacI-cfp/tetR-yfp,* Tn*5* P_{inR}-*echerry*	Derivatives of strain 5591 with a mini-Tn*5* P_{inR}-*echerry* insertion	This study
5605 5607 5608	UWC1-*clc*5, P1867-$tetO_{ARRAY}$, Δ*mfsR,* $lacO_{ARRAY}$, Tn*7 araC;lacI-cfp/tetR-yfp,* Tn*5* P_{inR}-*echerry*	Derivatives of strain 5592 with a mini-Tn*5* P_{inR}-*echerry* insertion	This study
5647	UWC1-*clc*5, Δ*oriT1,* $lacO_{ARRAY}$, Tn*7 araC;lacI-cfp*	Derivative of strain 5597 with the mini-Tn*7 araC*-P_{BAD}-*lacI-cfp* insertion	This study
5648	UWC1-*clc*5, Δ*oriT2,* $lacO_{ARRAY}$, Tn*7 araC;lacI-cfp*	Derivative of strain 5598 with the mini-Tn*7 araC*-P_{BAD}-*lacI-cfp* insertion	This study
5712	UWC1-*clc5* Δ*oriT1*, $lacO_{ARRAY}$, Tn*7 araC;lacI-cfp*, Tn*5* P_{inR}-*echerry*, Tn*5* compl-*oriT1*	Derivative of strain 5688 complementing deletion of *oriT1* in trans	This study
5713	UWC1-*clc5* Δ*oriT2*, $lacO_{ARRAY}$, Tn*7 araC;lacI-cfp*, Tn*5* P_{inR}-*echerry*, Tn*5* compl-*oriT2*	Derivative of strain 5673 complementing deletion of *oriT2* in trans	This study

1. McClure, N. C., Weightman, A. J. & Fry, J. C. Survival of *Pseudomonas putida* UWC1 containing cloned catabolic genes in a model activated-sludge unit. *Appl Environ Microbiol* **55**, 2627-2634 (1989).
2. Sentchilo, V. *et al.* Intracellular excision and reintegration dynamics of the ICE*clc* genomic island of *Pseudomonas knackmussii* sp. strain B13. *Mol Microbiol* **72**, 1293-1306 (2009).
3. Pradervand, N. *et al.* An operon of three transcriptional regulators controls horizontal gene transfer of the Integrative and Conjugative Element ICE*clc* in *Pseudomonas knackmussii* B13. *PLoS Genet* **10**, e1004441 (2014).
4. Delavat, F., Mitri, S., Pelet, S. & van der Meer, J. R. Highly variable individual donor cell fates characterize robust horizontal gene transfer of an integrative and conjugative element. *Proc Natl Acad Sci U S A* **113**, E3375-3383 (2016).
