# Supplementary material for: Transient Replication in Specialized Cells Favors Transfer of an Integrative and Conjugative Element
Source: mBio. 2019 Jun 11;10(3):e01133-19. doi: 10.1128/mBio.01133-19 (PMC6561031; doi:10.1128/mBio.01133-19)
Supplement: FIG S2 [file mBio.01133-19-sf002.pdf]

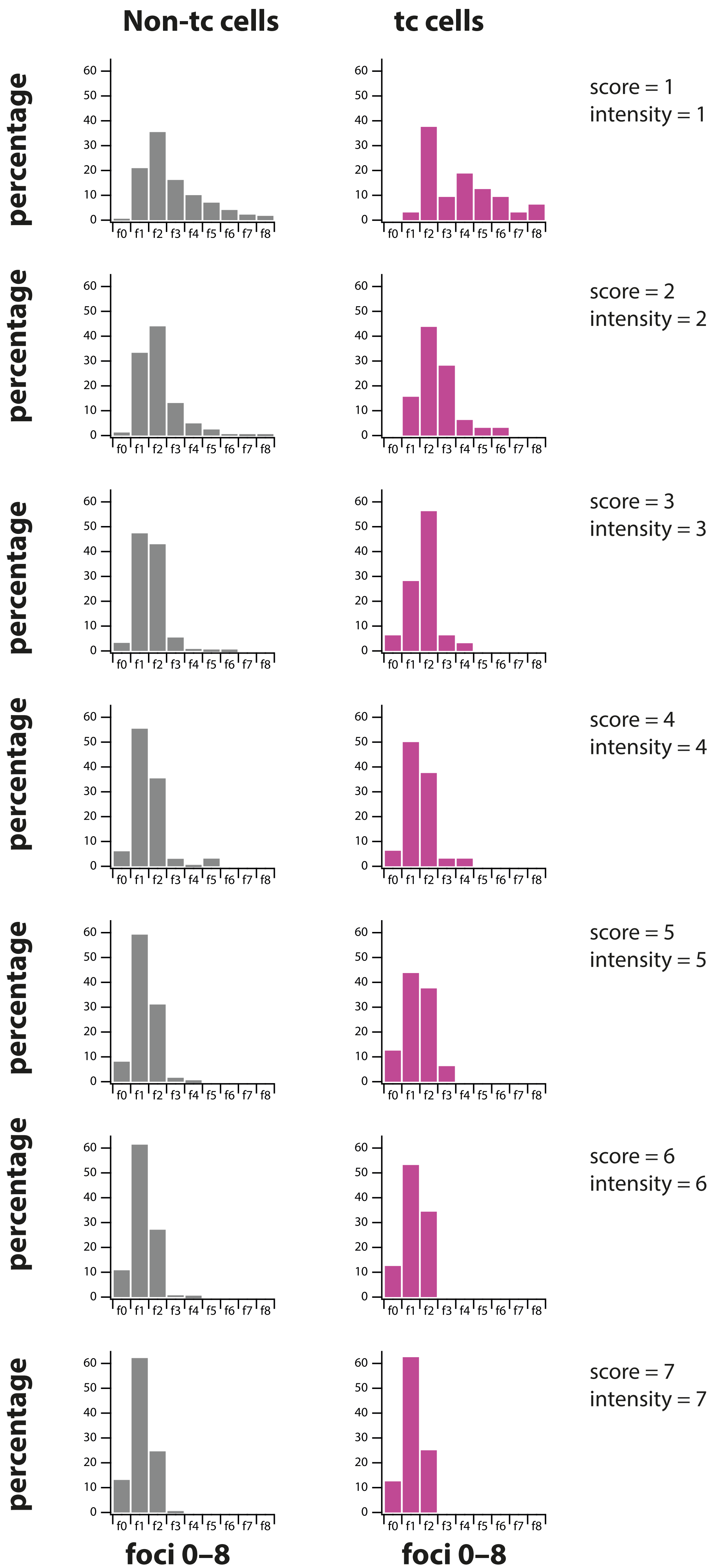
Non-tc cells
tc cells
percentage
foci 0–8
f0 f1 f2 f3 f4 f5 f6 f7 f8
0 10 20 30 40 50 60
score = 1
intensity = 1
score = 2
intensity = 2
score = 3
intensity = 3
score = 4
intensity = 4
score = 5
intensity = 5
score = 6
intensity = 6
score = 7
intensity = 7

Supplementary Figure S2 | Effect of score and intensity thresholding on foci distributions in *P. putida* UWC1-*clc5, ∆mfsR, ∆attL,* $lacO_{ARRAY}$*, Tn7 araC;lacI-cfp, Tn5* P_{inR}*-echerry* (strain 5357). Since this strain lacks the ICE attachment sites, it cannot excise, which was verified by PCR (Delavat, 2016). The lowest threshold setting with minimal foci appearance >2 (score=6 and intensity = 6) was thus used for the experiments

Delavat, F., Mitri, S., Pelet, S. & van der Meer, J. R. Highly variable individual donor cell fates characterize robust horizontal gene transfer of an integrative and conjugative element. Proc Natl Acad Sci U S A 113, E3375-3383 (2016).
