# Supplementary material for: Transient Replication in Specialized Cells Favors Transfer of an Integrative and Conjugative Element
Source: mBio. 2019 Jun 11;10(3):e01133-19. doi: 10.1128/mBio.01133-19 (PMC6561031; doi:10.1128/mBio.01133-19)
Supplement: FIG S3 [file mBio.01133-19-sf003.pdf]

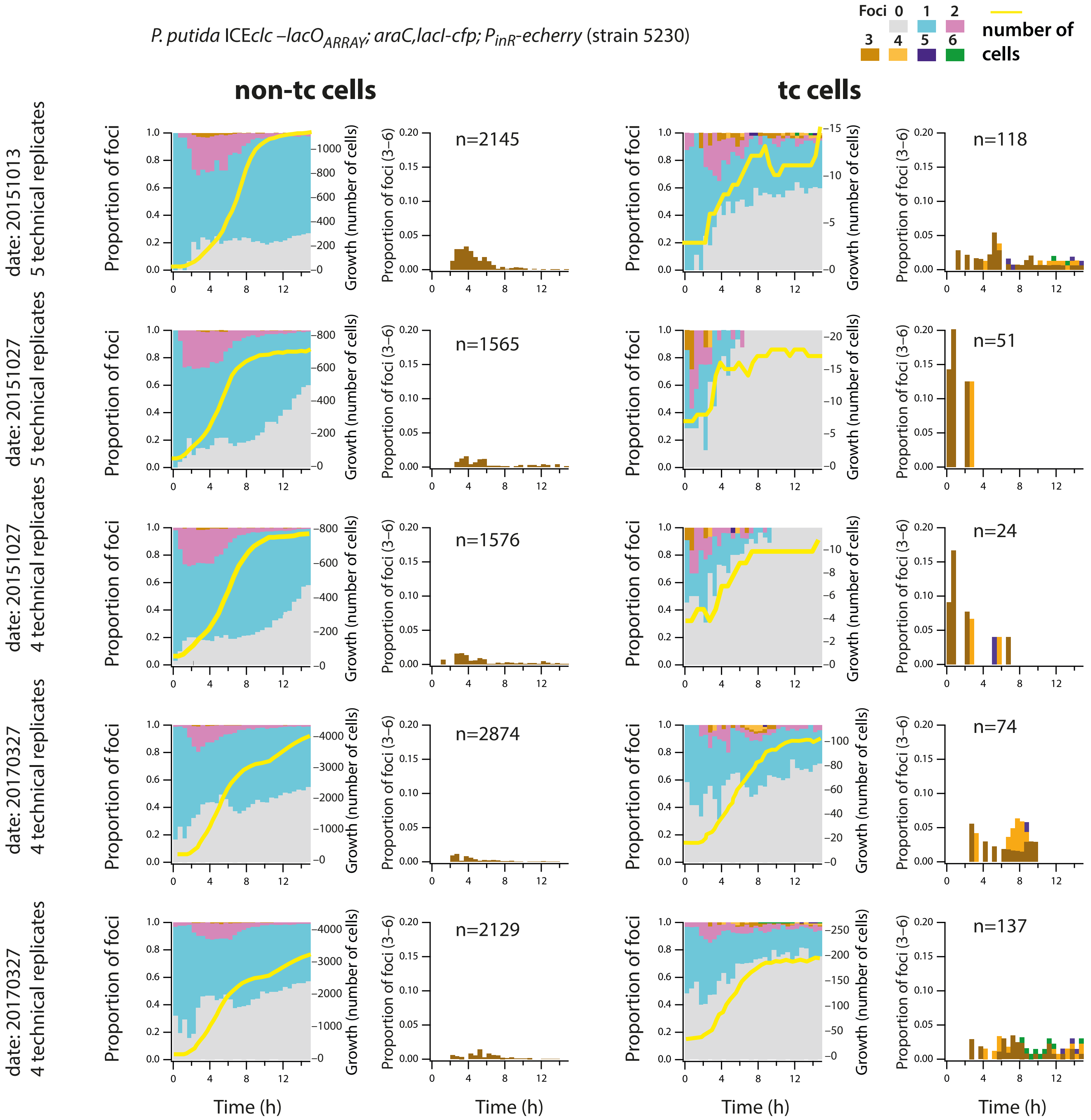

P. putida ICEclc –lacO ARRAY; araC,lacI-cfp; PinR-echerry (strain 5230)
Foci 0 1 2 3 4 5 6
number of cells
non-tc cells
tc cells
date: 20151013 5 technical replicates
date: 20151027 5 technical replicates
date: 20151027 4 technical replicates
date: 20170327 4 technical replicates
date: 20170327 4 technical replicates
Proportion of foci
Growth (number of cells)
Proportion of foci (3–6)
n=2145
n=118
n=1565
n=51
n=1576
n=24
n=2874
n=74
n=2129
n=137
Time (h)

**Supplementary Figure S3 |** Biological replicates of foci formation in non-tc and tc cells in *P. putida* ICE*clc-lacO*$_{ARRAY}$*; araC,lacI-cfp; P*$_{inR}$*-echerry* (strain 5230). Proportional foci distributions over time of growth as in Fig. 2 in the main text, for all quantified foci, or per category of foci ≥ 3. Numbers n= indicate the total amount of cells observed for each category during the duration of the experiment. Indications on the side indicate the number of technical replicates grouped per stack plot.
