# Supplementary material for: Transient Replication in Specialized Cells Favors Transfer of an Integrative and Conjugative Element
Source: mBio. 2019 Jun 11;10(3):e01133-19. doi: 10.1128/mBio.01133-19 (PMC6561031; doi:10.1128/mBio.01133-19)
Supplement: FIG S4 [file mBio.01133-19-sf004.pdf]

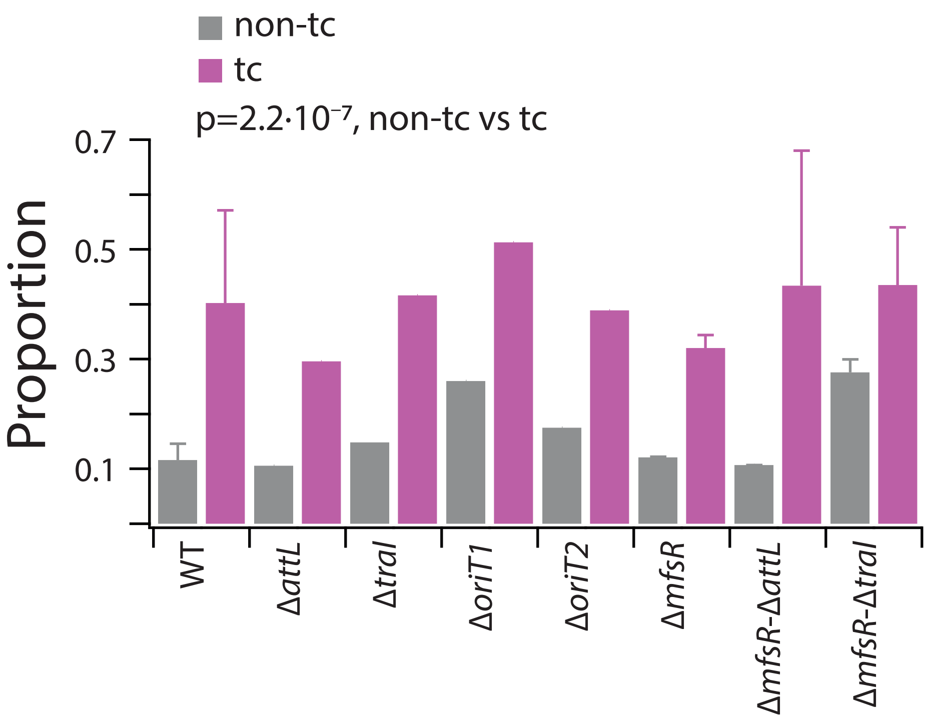

Supplementary Figure S4 |

Proportions of cells without any detectable foci in non-tc and tc cells of *P. putida* ICE*clc-lacO*$_{ARRAY}$*; araC,lacI-cfp*; P$_{inR}$-*echerry* with mutations in critical ICE excision or replication functions.

Error bars indicate calculated standard deviations from the mean of biological replicates. P-value of testing the proportions between non-tc and tc cells across all strains (single-sided t-test, hypothesis that tc cells have higher proportions of cells with any detected foci).
