# Supplementary material for: Transient Replication in Specialized Cells Favors Transfer of an Integrative and Conjugative Element
Source: mBio. 2019 Jun 11;10(3):e01133-19. doi: 10.1128/mBio.01133-19 (PMC6561031; doi:10.1128/mBio.01133-19)
Supplement: FIG S5 [file mBio.01133-19-sf005.pdf]

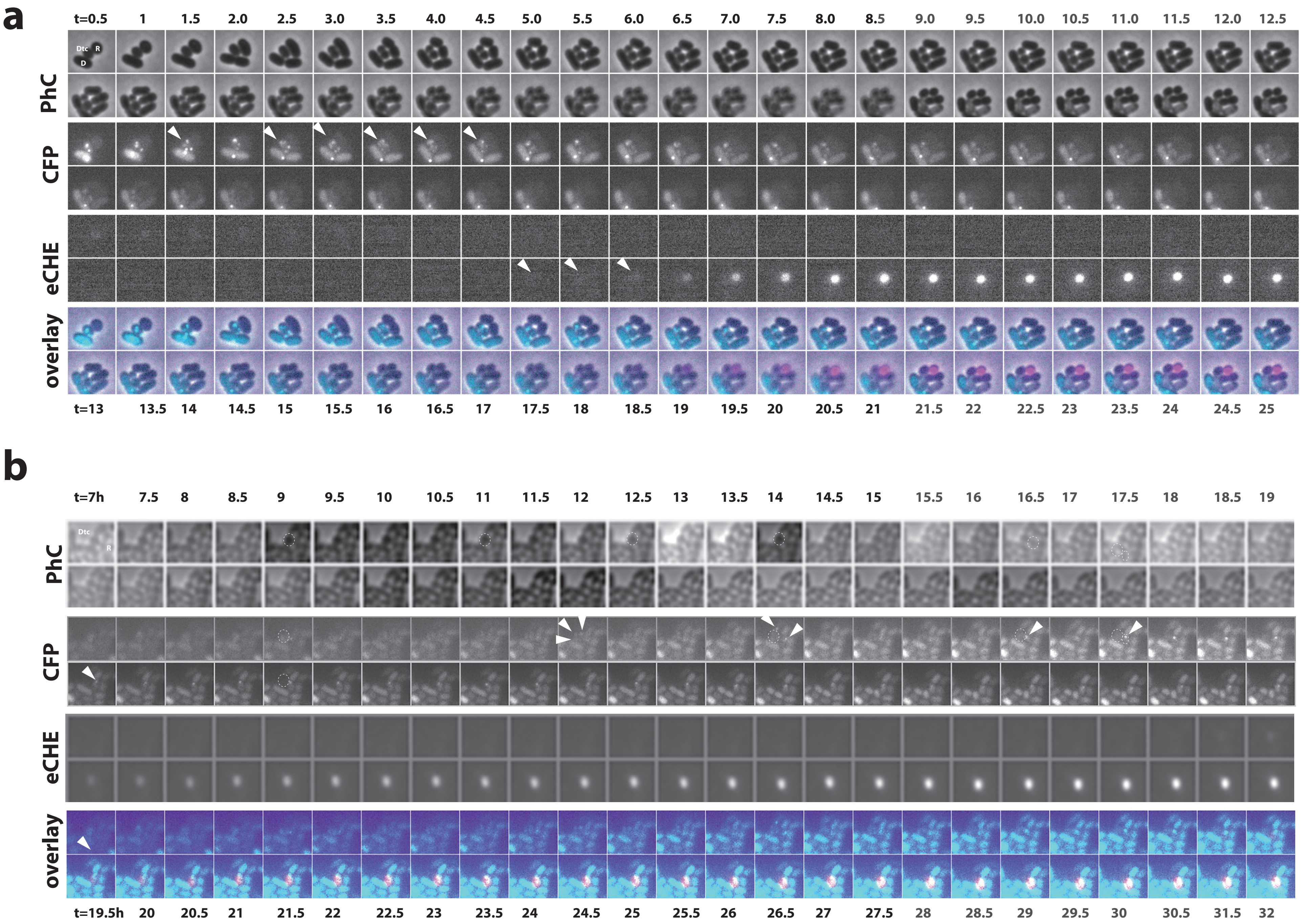
a
t=0.5
1
1.5
2.0
2.5
3.0
3.5
4.0
4.5
5.0
5.5
6.0
6.5
7.0
7.5
8.0
8.5
9.0
9.5
10.0
10.5
11.0
11.5
12.0
12.5
Dtc
R
D
PhC
CFP
eCHE
overlay
t=13
13.5
14
14.5
15
15.5
16
16.5
17
17.5
18
18.5
19
19.5
20
20.5
21
21.5
22
22.5
23
23.5
24
24.5
25
b
t=7h
7.5
8
8.5
9
9.5
10
10.5
11
11.5
12
12.5
13
13.5
14
14.5
15
15.5
16
16.5
17
17.5
18
18.5
19
Dtc
R
PhC
CFP
eCHE
overlay
t=19.5h
20
20.5
21
21.5
22
22.5
23
23.5
24
24.5
25
25.5
26
26.5
27
27.5
28
28.5
29
29.5
30
30.5
31.5
32

Supplementary Figure S5 | Full relevant time steps in ICE*clc* transfer between *P. putida* ICE*clc* Δ*mfsR* lac_{OARRAY} *lacI-cfp* (strain 5224) as donor and *P. putida* UWC1 *echerry*-'trap', *lacI-cfp* (strain 5248) as recipient. (Expanded data from Figure 5a and b in the main text). Time steps: 30 min. PhC, phase contrast. CFP, cyan fluorescent protein, eCHE, eCherry fluorescence. Overlay in **a**: PhC + CFP (cyan) + eCHE (magenta). Overlay in **b:** CFP (cyan) + eCHE (magenta).
